# Liposomal bupivacaine after reduction mammaplasty: is it worth the shot? A single-blind, breast-split randomized clinical trial

**DOI:** 10.1016/j.jpra.2026.06.003

**Published:** 2026-06-18

**Authors:** Sara M. Hussein, Lauren T. Gates-Tanzer, Brooke E. Willborg, Daniel E. Sotelo Leon, Samyd S. Bustos, Christin A. Harless, Uldis Bite, Vahe Fahradyan, Aparna Vijayasekaran, Jorys Martinez-Jorge, Nathan J. Brinkman, Ryan E. Hofer, Charles R. Sims, Thomas M. Stewart, Basel A. Sharaf

**Affiliations:** a Division of Plastic and Reconstructive Surgery, Department of Surgery, Mayo Clinic, 200 First St SW, Rochester, MN 55905, United States; b Department of Pharmacy, Mayo Clinic, 200 First St SW, Rochester, MN 55905, United States; c Department of Anesthesiology and Perioperative Medicine, Mayo Clinic, 200 First St SW, Rochester, MN 55905, United States; d Center for Aesthetic Medicine and Surgery, Mayo Clinic, 200 First St SW, Rochester, MN 55905, United States

**Keywords:** Bupivacaine, Breast reduction, Postoperative pain, Pain measurement, Local anesthesia, Analgesia

## Abstract

**Background:**

Reduction mammaplasty is a common procedure to relieve symptoms of macromastia. Amid the ongoing opioid crisis, improving postoperative pain control with judicious opioid prescribing practices has become a clinical priority. Liposomal bupivacaine (LB; Exparel) is a long-acting local anesthetic that may reduce opioid requirements, though evidence supporting its use in breast reduction is limited. This study evaluated the efficacy of LB in reducing postoperative pain following reduction mammaplasty.

**Methods:**

In this prospective, single-blind, breast-split controlled trial, 32 adult female patients undergoing bilateral reduction mammaplasty were enrolled at a single academic center after IRB approval. Each breast received standard bupivacaine (SB; 0.25% bupivacaine hydrochloride); one side was randomly assigned to receive additional LB. Pain scores were recorded using the Numerical Pain Rating Scale (0–10) twice daily on postoperative days (POD) 1 through 3. Pain scores between sides were analyzed using paired *t*-tests and Wilcoxon signed-rank tests.

**Results:**

The average patient age was 45.4 years (SD 14.4), with a mean BMI of 29.5 kg/m² (SD 4.3). No patients were lost to follow-up. Pain scores were lower on the LB side during POD1 and POD2. The greatest difference was noted on POD1 AM, with a mean reduction of -0.8 and median difference of -1 (p = 0.012). By POD3, pain scores equalized between sides. No adverse events or complications were observed.

**Conclusion:**

LB provided a modest reduction in pain scores during the first two postoperative days compared to standard bupivacaine. A more robust, adequately powered study is necessary to confirm the findings.

## Introduction

According to the 2023 American Society of Plastic Surgeons (ASPS) procedural statistics, reduction mammaplasty procedures increased by 7% compared to the previous year, ranking third in frequency after breast augmentation and breast lift.^1^, ^2^, ^3^, ^4^ This outpatient procedure has been shown to be both safe and cost-effective, with outcomes comparable to inpatient surgery.^5^, ^6^, ^7^ However, data on strategies to optimize recovery and pain control remain limited. Plain bupivacaine (0.25% bupivacaine hydrochloride) is commonly used for regional analgesia in postoperative pain management. Despite its efficacy, its relatively short half-life (6–12 h) necessitates repeated dosing or continuous infusion. In 2011, the U.S. Food and Drug Administration approved liposomal bupivacaine (LB; EXPAREL®), an extended-release formulation designed to provide analgesia for up to 96 h with a single injection.^8^^,^^9^ While some studies have shown LB to reduce opioid use, length of stay, and healthcare costs.^9^, ^10^, ^11^, ^12^, ^13^, findings have been inconsistent across surgical procedures.

Although LB has been studied in breast augmentation, mastectomy, and various other surgeries.^8^^,^^14^^,^^15^, its use in reduction mammaplasty remains underexplored. Existing retrospective data suggests LB may reduce postoperative pain, opioid consumption, and length of stay, particularly in patients with lower BMI and premenopausal status.^16^^,^^17^ However, conflicting results highlight the need for prospective evaluation. Of note, the dissection and incision pattern of a reduction mammaplasty is more significant, and thus our study provides data for efficacy in this patient population. Therefore, a prospective clinical trial was conducted to assess the efficacy of LB in enhancing postoperative pain control and recovery following reduction mammaplasty.

## Methods

### Study design and patient selection

**Fig. 1:**
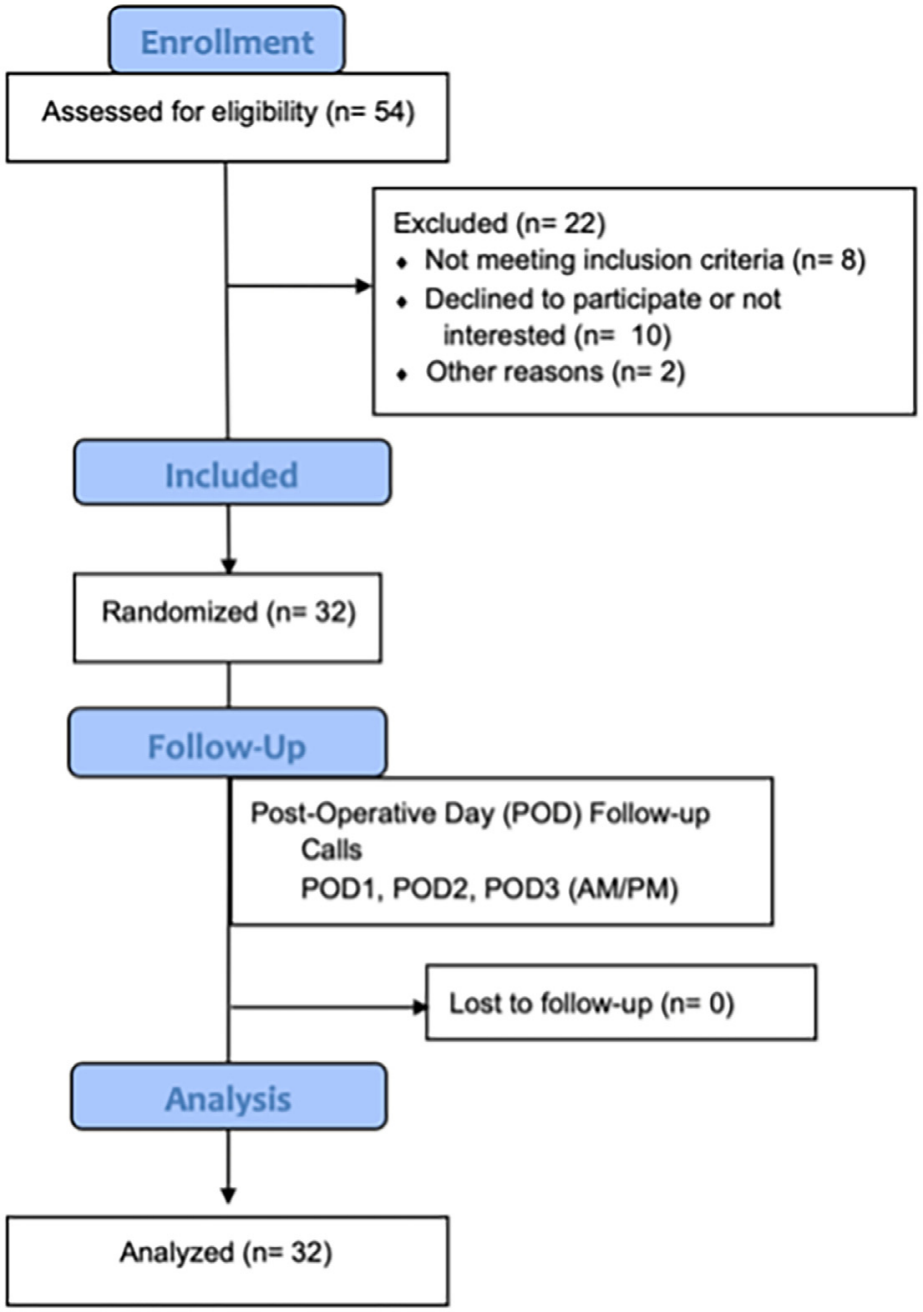
Consort Flow Diagram.

Following Institutional Review Board approval (IRB #: 21–007,358), a prospective, randomized, single-blind control trial was conducted on 32 patients with symptomatic macromastia undergoing reduction mammaplasty from January 2024 through March 2025 (Fig. 1**)**. Exclusion criteria included age <18 years, inability to consent, chronic pain requiring daily analgesics, pregnancy, concomitant non-breast surgical procedure, prior chest wall irradiation or breast surgeries, allergy to bupivacaine or liposomal bupivacaine, liver/kidney dysfunction, or anticoagulant use. Notably, no participants were lost to follow-up.

### Patient characteristics and pain management protocol

Baseline demographic and clinical characteristics, including age, race/ethnicity, body mass index (BMI), smoking status, menopausal status, and American Society of Anesthesiologists (ASA) physical status, were collected. Surgical variables collected included bra cup size (baseline and target), Schnur scale values, body surface area (BSA), resected breast weight, use of liposuction, and postoperative complications, if any. All patients received standard multimodal analgesia: acetaminophen, celecoxib (if not contraindicated), and opioids (e.g., oxycodone). Opioid consumption was tracked. Each patient received bupivacaine hydrochloride 0.25% (SB) bilaterally, with one breast randomized to also receive liposomal bupivacaine (LB; Exparel).

### Surgical techniques and pain medications administration

Procedures used either superomedial or inferior pedicle Wise-pattern reduction mammaplasty. Hemostasis was achieved with unipolar/bipolar electrocautery or hemoclips. Post-closure, both LB and SB were infiltrated into the subcutaneous tissue of one breast, while the other received SB, see **Appendix A,** using identical technique and volume (40 mL per breast; Table 1). Using a 22-gauge needle on a 20 mL syringe, the infiltration was performed along the horizontal limb of the Wise-pattern incision and the lateral chest wall. Participants were blinded to treatment allocation; the operating surgeon was aware of which side received LB as infiltration was performed by the surgeon intraoperatively. Discharge analgesia included acetaminophen 500 mg, celecoxib 200 mg, gabapentin, and opioids (oxycodone 5 mg or hydromorphone). Additionally, gabapentin 100–300 mg capsules with ibuprofen 200 mg tablets were included in the discharge plan for some patients.

**Table 1:** Composition of locoregional anesthetic solutions administered to the treated (LB) and control (SB) breasts.

	Treated (Liposomal Bupivacaine) Breast	Control (Standard Bupivacaine) Breast
Liposomal Bupivacaine	**266mg/20 mL**	0 mL
Bupivacaine 0.25%	**50mg/20 mL**	
Saline	0 mL	**20 mL**
Total volume	**40 mL**	**40 mL**

### Pain scores assessment

Postoperative pain was assessed using the validated Numeric Pain Rating Scale (NPRS), which ranges from 0 (no pain) to 10 (worst pain imaginable).^18^ The NPRS values were assessed at 24, 48, and 72 h postoperatively via twice-daily phone calls. Patients were asked to rate each breast independently, including five anatomical regions: medial, lateral, axillary, inframammary fold (IMF), and nipple-areolar complex (NAC). A ≥ 2-point difference was considered clinically meaningful per Karcioglu et al..^18^ Day-of-surgery scores were excluded due to variable discharge timing.

### Statistical analysis

Each patient served as their own control. Descriptive statistics were performed in BlueSky Statistics 10.3 (BlueSky Statistics, LLC, IL, USA), summarizing patient demographics and perioperative variables. Mean, standard deviation (SD), median, interquartile range (IQR) were used for continuous variables, such as age, body mass index (BMI), postoperative pain scores, and opioid consumption. This study includes paired comparison among the patients in split breast treatment group (i.e. comparison at a breast level). Sample size calculation reflected within patient comparison of pain ratings between breasts based on a pain rating scale from 0–10. Based on normal distribution assumption, we estimated the standard deviation of the within patient difference to be approximately 1.67 (i.e. 10/6) units, if patients utilize the entire pain range of 10 points. At a 5% level of significance, a standard deviation of 1.67 points, with a paired t-test with 80% power and 24 patients enrolled, a difference in pain score of 1 point could be detected between treated breasts, within a patient.

Comparisons were conducted using Wilcoxon signed rank test (Mann-Whitney U tests) for non-normally distributed variables and chi-square or Fisher’s exact tests for categorical data. Effect sizes were calculated using Cohen’s d for paired samples to estimate the magnitude of within-substance differences in pain reduction. Additionally, Pearson correlation coefficients were used to assess the strength and direction of associations between the variables, including patient characteristics, pain scores, and postoperative pain scores. A significance level of p < 0.05 was considered statistically significant. Data visualization and graphical representations were generated using Python (v3.8) and Matplotlib library to illustrate trends and comparisons effectively.

## Results

A total of 32 female patients undergoing bilateral reduction mammaplasty were enrolled in the study and included in the final analysis (Fig. 1). The average age was 45.4 years (SD=14.37), BMI was 29.5 kg/m^2^ (SD=4.3), and BSA was 1.92 (SD=0.15 m²). Most patients were healthy (ASA-II; n = 23, 74.2%). The most common preoperative breast cup size was DDD (29%) with 78% desiring a postoperative B/C cup. Median excised breast weight was 664 g on the LB side and 604 g on the control side, yielding a clinically nonsignificant difference (57 g; P < 0.48) and supporting the comparability of both sides. Bilateral axillary liposuction was performed in 11 patients (34.4%), with lipoaspirate volumes of 10–20 mL. Patient characteristics are summarized in **supplemental table 1**. Notably, tumescent solution containing lidocaine was only used in 7 patients (64% of those who underwent lateral chest wall liposuction), therefore, this is unlikely to confound baseline pain scores as its 1.5–2-hour half-life renders any analgesic effect negligible by POD1 AM assessments. Nineteen patients (61%) were discharged same-day; the rest stayed overnight.

### Pain score trends

**Fig. 2:**
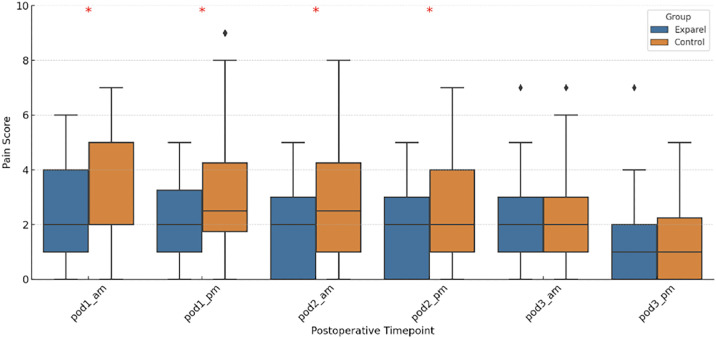
Distribution of Pain Scores by Treatment Side and Timepoint.

Postoperative pain scores were recorded twice daily over POD1-POD3 for both breasts, with patients blinded to the treatment side. Using both mean and median differences, the postoperative pain scores were compared between the LB-treated and control breasts (Tables 2 **and** 3). Due to non-normal distribution, non-parametric methods were prioritized (Table 3). The LB side consistently showed lower pain scores on POD1 and POD2 (Fig. 2).

**Table 2:** Summary of Pain Scores across the Three Postoperative Days (POD1-POD3).

Time points Post operative Day (POD)	Treatment/Control Sides	Mean (± SD)	Range (min-max)	Mean difference	95% Confidence Interval	*P*-value
POD1 AM:	LB Side	2.28 (±1.85)	0–6	-0.8 (-1.35, -0.24)		0.001*
	Control Side	3.07 (±2.04)	0–7			
POD1 PM:	LB Side	2.3 (±1.5)	0–5	-0.9 (-1.63, -0.08)		0.03*
	Control Side	3.1 (±2.3)	0–9			
POD2 AM	LB Side	2.0 (±1.74)	0–5	-0.9 (-1.7, -0.18)		0.018*
	Control Side	2.9 (±2.41)	0–8			
POD2 PM	LB Side	2 (±1.7)	0–5	-0.5 (-0.9, -0.03)		0.038*
	Control Side	2.5 (±2.2)	0–7			
POD3 AM	LB Side	1.9 (±1.6)	0–7	-0.25 (-0.71, 0.21)		0.27
	Control Side	2.1 (±1.7)	0–7			
POD3 PM	LB Side	1.5 (±1.5)	0–7	-0.1 (-0.5, 0.3)		0.63
	Control Side	1.6 (±1.6)	0–5			

⁎ T-test, Paired Samples.

**Table 3:** Comparison of Pain Scores Between the LB and Control Sides across the Three Postoperative Days (POD1-POD3).

Time points Post operative Day (POD)	Median differences	95% Confidence Interval	V	*P*-value
POD1 AM	-1	(1.5, -0.000002)	78	0.012*
POD1 PM	-1	(-1.9, -0.00007)	75	0.031*
POD2 AM	-1	(-2, -0.000004)	65.5	0.014*
POD2 PM	-1	(-1.5, -0.00004)	44	0.038*
POD3 AM	-0.7	(-1, 0.000057)	64	0.2
POD3 PM	−0.00005	(-1.5, 1)	43	0.56

⁎ Two-sided Wilcoxon signed rank test with continuity correction. Pseudo median is the reported median above.

Side-by-side box-and-whisker plots of NPRS pain scores on the LB and control SB sides at each postoperative timepoint. Boxes show the interquartile range (IQR; 25th-75th percentiles). * A red asterisk is placed above each timepoint where the Wilcoxon test showed statistical significance (p < 0.05) between sides.

On POD1 AM, the LB side demonstrated a mean difference of -0.8 (*p* *=* 0.001) and a median difference of -1 (*p* = 0.012). Similar differences persisted into POD1-PM and POD2-AM (mean -0.9, *p* = 0.03; median -1, *p* = 0.031). By POD2 PM, the mean pain difference narrowed to -0.5 (*p* = 0.038), though the median difference remained -1 (*p* = 0.038). By POD3, pain scores had leveled off between both sides. Differences were no longer statistically significant, with mean differences of -0.25 and -0.1 (*p* = 0.27) and median differences of -0.7 and -0.1, respectively (*p* > 0.05).

In our cohort, neither the BMI nor the weight of excised breast tissue demonstrated a positive correlation with postoperative pain scores or any observed trend. Twelve patients (37.5%) reported no narcotic use postoperatively. The median number of narcotic pills used, on the other hand, was 1 (IQR: 3.3), indicating minimal opioid use. Control side pain scores correlated with narcotic use on POD1 AM and POD3 PM (r = 0.51, 0.45), while LB side showed a notable correlation only on POD3 PM (r = 0.58). To ensure a comprehensive evaluation of potential confounding factors and to transparently account for variability in analgesic management, subgroup analysis examined preoperative analgesia and discharge prescriptions of ≥3 medications. No significant differences in pain scores were found across POD1–3 (**supplemental table 2**).

### Pain distribution

Pain location was assessed across breast quadrants. The IMF and axilla were most frequently reported (36% and 33%), likely due to drain placement. Axillary pain persisted across all time points. Medial, NAC, and lateral regions were less painful on the LB side. On the control side, medial and NAC pain fluctuated more, especially on POD3. Pain location did not significantly differ between sides and was more influenced by procedural factors.

## Discussion

This prospective single-blinded, split-breast clinical trial examined the effect of liposomal bupivacaine (LB) versus standard bupivacaine (SB) in reduction mammaplasty. The LB-treated side consistently demonstrated statistically significant but modest analgesic advantage during POD1–2, with a 1-point difference on a 11-point numeric pain rating scale (NPRS).^18^ However, differences did not meet the pre-specified 2-point threshold for clinical significance. By POD3, this benefit had faded, aligning with the known LB’s pharmacokinetic profile, providing 72 h of analgesia, with systemic plasma levels lasting 96 h after local administration.^8^

The postoperative pain can aggravate an already psychologically distressing experience and negatively impact quality of life.^19^ Over the last 2 decades, the introduction of multimodal pain management for enhanced recovery after surgery (ERAS) protocols represented a major advancement in postoperative surgical care.^20^ Particularly, LB (Exparel, Pacira BioSciences, Tampa, Florida) has been advocated to improve post-operative analgesia in plastic surgical procedures since its FDA approval in 2011.^12^ Although LB was introduced to aesthetic breast surgery over a decade ago, few controlled trials have evaluated its use in breast reduction. The 2016 prospective trial by Nadeau et al. in breast augmentation was among the earliest efforts. Our study fills this gap using a rigorous intra-patient design, where each patient served as their own control, minimizing confounding factors such as pain threshold, systemic drug metabolism, and psychological perception. This design allowed for a direct comparison of localized analgesic effects.

### Discharge trends and hospital stay

Reduction mammaplasty is often performed as an outpatient procedure, though discharge practices vary. In our cohort, 61% were discharged the same day, while others stayed overnight based on the patient’s preference and convenience. While hospital stay was not a primary endpoint, our findings align with prior studies. For example, Jablonka et al. reported shorter stays (2.7 days vs. 4.1 days in control) in 128 patients receiving the Transversus Abdominis Plane (TAP) LB blocks during abdominal-based breast reconstruction, suggesting that effective regional analgesia may support earlier discharge.^21^ Though these findings involve ultrasound-guided percutaneous regional techniques and cannot be directly extrapolated to surgeon-administered wound infiltration as performed in this study.

### Pain score trends and opioid-sparing effect

Our results are consistent with some prior studies but diverge from others. A systematic review by Hamilton et al. found LB superior to placebo but not consistently better than SB across various procedures ^22^. Procedures in that review included knee arthroplasty, inguinal hernia, and bunionectomy, and among others. In reduction mammaplasty, Kalaria et al. reported reduced pain scores, antiemetic and opioid use in patients receiving LB.^16^, particularly in those with lower obesity class. However, less efficacy was observed in more obese or postmenopausal populations, possibly due to increased adipose tissue uptake.^16^ In our study, there is no correlation between pain scores and BMI. In augmentation mammaplasty, a prospective randomized trial by Nadeau et al..^11^ demonstrated statistically significant reduced pain scores with LB versus standard bupivacaine, but 70% of patients did not perceive the difference as clinically meaningful, raising questions about its cost-effectiveness in aesthetic applications.^11^ That study used instillation into the implant pocket rather than incision infiltration, which may explain the limited effect.^11^ Also, their maximum pain scores did not exceed 6/10, whereas our control side reached 9/10, indicating a more painful baseline and greater potential for analgesic impact.^11^^,^ ^23^

Other studies have shown mixed results. Lee et al. showed that LB parasternal intercostal block for sternotomy had slightly lower pain scores over 72 h but resulted in no difference in opioid consumptions and other secondary outcomes.^14^ On the other hand, recent evidence by Thuppal et al. demonstrated no significant difference in postoperative pain, narcotic consumption, or length of stay between patients receiving LB and SB during minimally invasive lung lobectomy ^24^.

Although a 2-point difference on the NPRS is frequently regarded as clinically significant in acute postoperative pain.^18^, smaller reductions may still be meaningful when associated with reduced opioid use, improved comfort, and faster recovery.^9^^,^^25^, ^26^, ^27^ Abdelsattar et al. reported that LB infiltration in implant-based reconstruction resulted in delayed opioid request, and reduced pain scores when compared to the paravertebral blocks.^28^ Batdorf et al. and Jablonka et al., who incorporated LB into their ERAS protocols, reported a 71% reduction in opioid consumption and shorter hospital stays.^20^^,^^21^ Although these literature findings support the premise that LB can provide locoregional analgesic effect and enhance patient comfort in the early postoperative period, supporting the opioid-sparing effect, the overall opioid utilization in our cohort was low, with 37.5% of patients reporting no narcotic use. Among those who did require opioids, the median number of pills consumed was only 1. Although correlations between pain scores and opioid use were observed, these did not reach statistical significance, and the study was not powered to detect differences in opioid-related outcomes. These findings suggest that while LB may contribute to improved early postoperative comfort, its independent opioid-sparing effect remains uncertain in our study setting.

Since our patients served as their own controls, the pain perception was measured separately in the treated vs. control breast, as the influence of systemic medications (e.g., the administered pre-op and discharge medications) would theoretically affect both sides equally.

### Anatomical pain distribution

Pain was most frequently reported in the axillary and inframammary fold (IMF) regions, likely due to drain placement rather than tissue manipulation. Axillary pain persisted across all time points. LB-treated breasts showed less frequent pain in the medial, NAC, and lateral regions. These findings suggest that targeted infiltration into high-pain anatomical zones may further optimize outcomes. No complications were observed in patients receiving LB or SB, supporting the safety of both agents.

Pectoral nerve (PECs I/II) blocks have recently been applied in breast reduction, with studies showing reduced perioperative opioid use with LB, though pain scores are not consistently improved.^17^ While PECs blocks represent a valid regional strategy, our institution reserves them primarily for oncologic breast procedures with axillary dissection. In reductions mammoplasty, patients receive general anesthesia with multimodal systemic analgesia, and surgeon-delivered LB infiltration remains our standard due to its efficiency, reproducibility, and minimal workflow disruption.

### Cost-Effectiveness considerations

The high upfront cost of LB remains a concern. Thuppal et al. reported significantly increased pharmacy costs with LB compared to SB ($1052 vs $596).^24^ Similarly, Nadeau noted the cost difference per-vial ($285 for LB vs. just over $1 for SB).^11^ Butz also observed no immediate cost savings when comparing LB to traditional pain pumps.^9^ This data questions the cost justification of integrating LB in settings where its benefits are not clearly offset by reductions in other healthcare costs.

However, other studies claimed potential downstream savings. Little et al. found lower total and direct hospital costs, shorter stays, and fewer readmissions with LB. Motakef et al. showed significant reductions in opioid and benzodiazepine use, length of stay, and billing charges.^29^ Similarly, Bajaj et al. reported favorable cost-effectiveness of TAP blocks in ERAS protocols.^13^ In such complex inpatient settings, LB may be economically justified by improved recovery and reduced resource utilization.

Local anesthetic adjuncts, including dexamethasone, have been shown in the literature to extend the analgesic duration of plain bupivacaine at substantially lower cost than LB. These have not been studied in reduction mammaplasty and represent a good opportunity for future cost-effectiveness trials.^32^ Nonetheless, a modest 1-point improvement on the NPRS scale in the early postoperative period warrants further evaluation. It remains to be answered whether the utility of LB in more anxious patients or patients with lower pain thresholds justifies its cost or whether LB should be routinely included in multimodal pain management of reduction mammaplasty patients.

### Limitations

Our study’s strengths include the split-breast design, randomized nature which minimizes inter-patient variability ^31^, and the use of both parametric and non-parametric analyses to validate findings. However, limitations include its single-center, and small sample size. The study was powered primarily for pain score differences, not secondary outcomes like hospital stay or opioid use. Overnight admission decisions were based on patient preference rather than clinical necessity. Additionally, we acknowledge methodological limitations in the effective dilution of bupivacaine in the control arm. To maintain equal injection volumes (40 mL per side) and to remain within the manufacturer-recommended maximum combined bupivacaine dose, 20 mL of normal saline was added to the control side, resulting in an effective SB concentration of 0.125% rather than 0.25%. Although equal volumes were used to prevent confounding by tissue distension, this concentration inequality may have contributed to the observed pain difference. However, LB contains a small immediately bioavailable fraction of free bupivacaine at the time of injection (approximately 3%). As a result, the estimated initial free-bupivacaine concentration between groups at time zero was similar despite the diluted control arm. Cost-related outcomes were beyond the scope of this study. The twice-daily assessment schedule, while pragmatic, captures limited intraday variation. Additionally, variability in postoperative pain may be influenced by unmeasured patient-level factors such as anxiety and pain sensitivity. Prior research by Katz et al. has shown that preoperative anxiety and emotional instability can significantly impact pain perception regardless of treatment modality.^30^ Lastly, bilateral pain rating may be subject to anchoring effects, as pain perception is partially a systemic phenomenon. Given that opioid use was assessed via patient self-report without granular quantification of dosing or timing, which may limit precision in evaluating opioid consumption patterns, future prospective trials should incorporate the downstream endpoints of opioid consumption and functional recovery to more comprehensively assess the clinical utility and opioid-sparing potential of LB in reduction mammaplasty.

## Conclusion

This prospective, single-blinded, single-split controlled clinical trial demonstrates a statistically significant but clinically modest reduction of 1 point on the NPRS in the LB-treated breast during POD1–2. This difference did not reach the pre-specified 2-point threshold for clinical significance. Pain localization patterns did not show any difference between the LB and control sides, with axillary and IMF regions being the most frequently reported pain locations, as expected. No complications related to LB were observed. In healthcare settings where the cost of LB may be prohibitive, our study advocates for the use of standard Bupivacaine as a part of a multimodality enhanced recovery protocol. LB may offer a slight clinical benefit over SB on POD 1 and 2, yet it is statistically significant. The benefit of LB vs SB administration when multiple surgical sites are involved (i.e. mommy makeover) warrant further study. Importantly, future adequately powered studies incorporating opioid consumption, functional recovery, and cost-effectiveness are needed.

## Funding

This study was supported by the Division of Plastic Surgery, Department of Surgery, Mayo Clinic. No external funding was received.

## Institutional review board approval/ethical approval

The study protocol received approval from our Institutional Review Board (IRB) under the reference number **21–007,358**. All patients provided written informed consent.

## Clinical trial URL

https://clinicaltrials.gov/study/NCT05891613?locStr=Rochester,%20MN&country=United%20States&state=Minnesota&city=Rochester&term=Liposomal%20Bupivacaine&rank=1.

## Authors’ contributions

As a senior author, BAS initiated the study, led its conception and design, oversaw patient recruitment, data collection, data and figure interpretation, manuscript drafting and critical revision, and provided senior oversight for all aspects of the project. Sara M. Hussein, Lauren T. Gates-Tanzer and Brooke Willborg contributed to study design, patient recruitment, data collection, data interpretation, and manuscript drafting and revision. Sara M Hussein performed the data analysis and consulted the Mayo Clinic Center for Clinical and Translational Science (CCaTS) for statistical review. Daniel E. Sotelo Leon and Samyd Bustos contributed to patient recruitment and revising the manuscript. Christin A. Harless, Uldis Bite, Vahe Fahradyan, Aparna Vijayasekaran, and Jorys Martinez-Jorge served as operating surgeons, recruited patients, contributed to data collection, and revising the manuscript. Nathan J. Brinkman provided pharmacologic expertise on anesthetic dosing and safety and early protocol development, while Ryan E. Hofer, Charles R. Sims III, and Thomas M. Stewart contributed anesthetic expertise, perioperative care, and manuscript review.

## Presenting at

Plastic Surgery The Meeting (PSTM) 2025 in New Orleans, October 9–12, 2025.

## Declaration of competing interest

The authors report no personal financial conflicts of interest. This study was supported by the Plastic Surgery Division, Department of Surgery, Mayo Clinic, and all investigators are employees of the Mayo Clinic. The Mayo Clinic has an institutional financial collaboration with Pacira BioSciences; however, no funds from Pacira BioSciences were used for this study, and the company had no role in the conception, design, conduct, analysis, or reporting of this study.

## Appendix Supplementary materials

Image, application 1

Supplemental Tables Legends Image, application 2
