# Supplementary material for: Liposomal bupivacaine after reduction mammaplasty: is it worth the shot? A single-blind, breast-split randomized clinical trial
Source: JPRAS Open. 2026 Jun 18;51:169–79. doi: 10.1016/j.jpra.2026.06.003 (PMC13355732; doi:10.1016/j.jpra.2026.06.003)
Supplement: Supplementary file 1 [file mmc1.docx]

| **Characteristics** | | **No. of patients or measurements** |
| --- | --- | --- |
| Sex | Female | *N= 32* |
| Race | White | *N= 30* |
|  | Black | *N= 1* |
|  | Asian | *N= 1* |
| Height | Mean in cm (SD) | 164.9 (6.45) |
| Weight | Mean in kg (SD) | 80 (11) |
| Body Mass Index (BMI) | Mean in kg/m^2^ (SD) | 29.5 (4.3) |
| Menopause | Yes | *N= 10* |
|  | No | *N= 22* |
| Liposuction | Yes | *N= 11* |
| Body Surface Area (BSA) | Mean in m^2^ (SD) | 1.93 (0.17) |
| Preoperative  Main Breast Cup | C | *N= 1* |
|  | D | *N= 3* |
|  | DD | *N= 4* |
|  | DDD | *N= 9* |
|  | E | *N= 1* |
|  | EE | *N= 1* |
|  | F | *N= 1* |
|  | H | *N= 3* |
|  | K | *N= 1* |
|  | Others | *N= 7* |
| Target Breast Cup | B | *N= 5* |
|  | B-C | *N= 8* |
|  | C | *N= 12* |
|  | C-D | *N= 2* |
|  | DD | *N= 1* |
|  | Others | *N= 4* |
| American Society of Anesthesiologists (ASA) physical status | ASA I: | *N= 7* |
|  | ASA II: | *N= 23* |
|  | ASA III: | *N= 2* |
|  | ASA IV: | *N= 0* |

**Supplemental table 1:** Summary of Patients’ Characteristics
