# Supplementary material for: Liposomal bupivacaine after reduction mammaplasty: is it worth the shot? A single-blind, breast-split randomized clinical trial
Source: JPRAS Open. 2026 Jun 18;51:169–79. doi: 10.1016/j.jpra.2026.06.003 (PMC13355732; doi:10.1016/j.jpra.2026.06.003)
Supplement: Supplementary file 2 — Supplemental Tables Legends [file mmc2.docx]

|  | Pre-op medications and ≥3 discharge medications | Mean | Std Deviation |
| --- | --- | --- | --- |
| In-Hospital Averaged Pain Level | No | 3.23 | 1.9253 |
|  | Yes | 3.2318 | 1.6305 |
| Treated_Pod1_AM | No | 2.15 | 1.9156 |
|  | Yes | 2.3409 | 1.8605 |
| Control_Pod1_AM | No | 2.75 | 2.0983 |
|  | Yes | 3.2273 | 2.0397 |
| Treated_Pod1_PM | No | 2.45 | 1.6406 |
|  | Yes | 2.1818 | 1.5004 |
| Control_Pod1_PM | No | 2.9 | 2.4244 |
|  | Yes | 3.2273 | 2.3489 |
| Treated_Pod2_AM | No | 2.1 | 1.9692 |
|  | Yes | 1.9545 | 1.6755 |
| Control_Pod2_AM | No | 2.6 | 2.2706 |
|  | Yes | 3.0909 | 2.5054 |
| Treated_Pod2_PM | No | 1.6 | 1.4298 |
|  | Yes | 2.1591 | 1.8605 |
| Control_Pod2_PM | No | 1.9 | 2.1318 |
|  | Yes | 2.7273 | 2.1587 |
| Treated_Pod3_AM | No | 1.4 | 1.3499 |
|  | Yes | 2.0909 | 1.6877 |
| Control_Pod3_AM | No | 1.8 | 1.8738 |
|  | Yes | 2.2727 | 1.6671 |
| Treated_Pod3_PM | No | 1 | 1.1547 |
|  | Yes | 1.6818 | 1.6729 |
| Control_Pod3_PM | No | 1.3 | 1.6364 |
|  | Yes | 1.6818 | 1.6729 |
| Narcotic Pills | No | 1 | 1.5635 |
|  | Yes | 2.5455 | 2.9069 |

**Supplemental table 2:** Mean postoperative pain scores (POD1–3, AM and PM) and narcotic consumption stratified by preoperative analgesia and ≥3 discharge pain regimen (Yes/No).
